# The effect of real-time CPR feedback and post event debriefing on patient and processes focused outcomes: A cohort study: trial protocol

**DOI:** 10.1186/1757-7241-19-58

**Published:** 2011-10-18

**Authors:** Gavin D Perkins, Robin P Davies, Sarah Quinton, Sarah Woolley, Fang Gao, Ben Abella, Nigel Stallard, Matthew W Cooke

**Affiliations:** 1 Heart of England NHS Foundation Trust, Bordesley Green East, Birmingham, B9 5SS, UK; 2 University of Warwick, Warwick Medical School, Gibbet Hill Road, Coventry CV4 7AL, UK; 3 Center for Resuscitation Science and Department of Emergency Medicine, University of Pennsylvania, Philadelphia, PA 19104, USA

**Keywords:** cardiac arrest, cardiopulmonary resuscitation, defibrillation, emergency medicine, guideline adherence, quality, resuscitation

## Abstract

**Background:**

Cardiac arrest affects 30-35, 000 hospitalised patients in the UK every year. For these patients to be given the best chance of survival, high quality cardiopulmonary resuscitation (CPR) must be delivered, however the quality of CPR in real-life is often suboptimal. CPR feedback devices have been shown to improve CPR quality in the pre-hospital setting and post-event debriefing can improve adherence to guidelines and CPR quality. However, the evidence for use of these improvement methods in hospital remains unclear. The CPR quality improvement initiative is a prospective cohort study of the Q-CPR real-time feedback device combined with post-event debriefing in hospitalised adult patients who sustain a cardiac arrest.

**Methods/design:**

The primary objective of this trial is to assess whether a CPR quality improvement initiative will improve rate of return of sustained spontaneous circulation in in-hospital-cardiac-arrest patients. The study is set in one NHS trust operating three hospital sites. Secondary objectives will evaluate: any return of spontaneous circulation; survival to hospital discharge and patient cerebral performance category at discharge; quality of CPR variables and cardiac arrest team factors. Methods: All three sites will have an initial control phase before any improvements are implemented; site 1 will implement audiovisual feedback combined with post event debriefing, site 2 will implement audiovisual feedback only and site 3 will remain as a control site to measure any changes in outcome due to any other trust-wide changes in resuscitation practice. All adult patients sustaining a cardiac arrest and receiving resuscitation from the hospital cardiac arrest team will be included. Patients will be excluded if; they have a Do-not-attempt resuscitation order written and documented in their medical records, the cardiac arrest is not attended by a resuscitation team, the arrest occurs out-of-hospital or the patient has previously participated in this study. The trial will recruit a total of 912 patients from the three hospital sites.

**Conclusion:**

This trial will evaluate patient and process focussed outcomes following the implementation of a CPR quality improvement initiative using real-time audiovisual feedback and post event debriefing.

**Trial registration:**

ISRCTN56583860

## Background

Each year approximately 30-35, 000 people sustain a cardiac arrest in hospitals in the UK. National audits in the UK and US report an initial survival rate of 50-60%[1,2]. Morbidity and risk of death are high in the first few days after cardiac arrest, however, after this high risk period has passed, the majority (80%) of people are relatively free from on-going morbidity and are alive at one year.

The International Liaison Committee for Resuscitation (ILCOR) have developed evidence based guidelines for resuscitation which are used across NHS trusts [3]. However these can only improve outcomes if they are successfully implemented into clinical practice [4].

The importance of the quality of CPR has been reinforced in a series of observational studies in humans. Chest compression depth [5,6]; rate [7]; ventilation rate [8] and duration of pre-shock pauses [6,9] have all been shown to influence the likelihood of a successful resuscitation attempt. Despite these compelling data, observational studies during real life resuscitation attempts consistently demonstrate sub-optimal implementation of resuscitation guidelines in practice [10,11].

The integration of real-time audio/visual feedback during actual CPR improves the quality of CPR in pre-hospital resuscitation attempts [5,12], but it's effect in hospital has been less clearly demonstrated [13]. The use of post event debriefing in simulator based CPR training significantly improves team performance [14,15] and is widely used in military and aviation practices for improving future performance. Junior doctors have also reported feeling un-prepared and concerned about managing cardiac arrest and have called for more feedback on their performance [16]. Systems of post-event debriefing of the cardiac arrest team of real life resuscitation atempts were associated with improved: knowledge; adherence to guidelines; quality of CPR and a significantly increase in survival (44% to 60%, P = 0.03)[17].

This promising study has some limitations and it is unclear if these findings could be directly extrapolated to clinical practice. Firstly, the historical control group preceded a major change in resuscitation guidelines (Guidelines 2005), so it is unclear if the improvement in survival was contaminated by the change in practice. Secondly, the casemix in this study are significantly different from UK practice (63% of arrests occurred in a critical care area as opposed to 11% in our own Trust and 15% nationally). Thirdly, CPR feedback technology was already in widespread use with the control cohort, which is rare in the UK at present.

In the recent ILCOR knowledge gaps and research prioritisation exercise, the paucity of evidence in this area was identified and the need for further research on feedback during and after CPR was prioritised [18]. The aim of this project is to test whether implementation of a CPR quality initiative (comprising of real-time CPR feedback technology supplemented with post-event debriefing) affect patient and process focused outcomes.

## Methods/Design

### Trial Approvals and Conduct

The trial is approved by the Coventry Research Ethics committee. The trial is registered on the International Standard Randomised Controlled Trial Registry (ISRCTN56583860). It will be carried out in accordance with the Medical Research Council (MRC) Good Clinical Practice Guidelines [19], applicable UK legislation and the Standard Operating Procedures of the Department of Anaesthesia, Critical Care, Pain and Resuscitation. The sponsor organisation for the trial is Heart of England NHS Foundation Trust. The trial is funded by the National Institute for Health Research (NIHR) Research for Patient Benefit (RfPB) Programme. The full trial protocol can be found in Additional File 1.

### Outcome Measures

The primary outcome for the trial is intial cardiac arrest survival (defined as sustained (> 20 minutes) return of spontaneous circulation). The secondary outcomes are split into two categories; patient focused and process focused outcomes. The patient focused outcomes include: any return of spontaneous circulation; survival to hospital discharge and cerebral performance category of patients at discharge. The process focussed outcomes focus on both quality of CPR and team factors. Quality of CPR outcomes are: quality of 2222 call; chest compression depth; chest compression rate; no-flow time; no-flow time adjusted; duration of pre-shock and post shock pause; ventilation rate; time to first shock (if initial rhythm VF/VT) and appropriate decision to shock. Team factors are; CPR knowledge amongst the cardiac arrest team and confidence/preparedness.

### Eligibility Criteria

Consecutive hospitalised adult patients who sustain a cardiac arrest during their hospital stay will be eligible for inclusion in the study according to the following criteria.

Individual patients will be eligible if:

1. The patient is known or believed to be aged 18 years or over.

2. The patient sustains a cardiac arrest and resuscitation is attempted (defined as loss of a pulse requiring the delivery of chest compressions).

Exclusion criteria will be:

1. If the patient has a Do-not-attempt resuscitation order written and docummented in their medical records

2. The cardiac arrest is not attended by a resuscitation team

3. If the cardiac arrest occurs out-of-hospital

4. Previous participation in this study

### Power and Sample Size

The sample size estimation is based on a baseline return of spontaneous circulation rate of 44% (from the last 12 month audit at the Trust). To predict a 16% absolute improvement in ROSC rate (as seen in the Edelson et al [17] study which had a similar baseline rate as our Trust) 152 patients will be required in each arm to achieve 80% power at a significance level of 0.05.

Secondary outcomes: Based on the data from the Edelson study and our own preliminary studies (n = 6); the study will have sufficient power (80%) at a significance level of 0.01 to detect the following improvements in CPR quality performance data (all figures are relative changes from baseline): chest compression depth (8%); ventilation rate (12%); no flow fraction (18%); appropriate decision to shock (15%).

### Sample Size Feasibility

The number of documented cardiac arrests across the 3 in-patient hospital sites was recorded as 651 in 2007 (Heartlands 297; Solihull 168; Good Hope 186). We propose to run the control phase and intervention phase at Heartlands and Good Hope for 13 months each. Patients sustaining a cardiac arrest on more than one occasion were not included in this audit. Cardiac arrest event data are used as a quality indicator by the Trust, so based on our current experience we anticipate that the data informing the primary outcome of the study (survived event) will be complete. Although we anticipate capturing all eligible cardiac arrests, we have based our recruitment windows assuming an 80% enrollment rate at the smallest centre (Solihull).

### Consent

Prospective consent from research participants prior to enrolment is impossible in this trial; the occurrence of a cardiac arrest is unpredictable, and a victim becomes unconscious within seconds. Treatment (in the form of CPR) must be started immediately in an attempt to save the person's life. It is therefore not practical to consult a carer or independent clinician without causing the potential participant harm as a result of delaying treatment. Conducting research in emergency situations where a patient lacks capacity is regulated by the Mental Capacity Act (2005) in England and Wales and the Adults with Incapacity Act (2000) in Scotland. The relevant ethics committees have determined that the research methods are compliant with the requirements of this legislation.

Process and outcome data are routinely recorded on the Trust clinical CPR audit database. Data for analysis for research purposes will be extracted and anonomised from this database.

Regretably the nature of the emergency in question mean that the majority (> 80%) of resuscitation attempts will be unsuccessful. Our experience of approaching relatives in the hours and days after a cardiac arrest has been that this causes distress and confusion at a time when they are already burdended by either the loss of a loved one or shock of a sudden deterioation in health status. Our assessment of the burdens of approaching relatives or patients for consent to use anonymised data which are already routinely collected outweigh the benefits.

### Protection against Bias

It is not possible to blind either the cardiac arrest team or investigator team from the study intervention. The following steps will be taken to protect against bias. The primary and secondary patient focused outcomes are objective outcomes that cannot be influenced by knowledge of the treatment allocation. The CPR performance and electrocardiographic data are collected electronically directly from the study defibrillators after resuscitation attempts and are therefore not subject to bias. Researchers measuring parameters requiring interpretation from Q-CPR (% correct shock decisions, pre and post-shock pauses) will be blinded from knowing whether the data are from the control or intervention site.

### Trial Intervention/Treatments

This study will be a prospective, cohort study. The study will evaluate two interventions: real-time audio-visual feedback during CPR and real-time audio-visual feedback during CPR plus post event feedback using the Q-CPR system (described below). We have not included a 3rd arm (post event feedback alone) as this would be unlikely to be used in clinical practice as to be able to provide this model of post event feedback requires the use of the Q-CPR equipment, which incorporates the real-time audiovisual feedback facility.

The study will be divided into 2 phases (see table 1). During the first phase, baseline data will be collected at each site without any intervention. During the second phase, real-time feedback phase will be implemented at one site (Good Hope-GH) and real-time audiovisual feedback plus post event debriefing at one site (Heartlands-BHH). The remaining site (Solihull-SH) will act as the control site during both interventions. The purpose of the control site is to exclude any Trust-wide or other temporal changes in care as potential explanations for any changes in outcomes at Heartlands/Good Hope.

**Table 1:** Showing the two phases and interventions at the 3 hospital sites

Site	Phase 1	Phase 2
**GHH**	Control	Realtime audiovisual feedback only

**BHH**	Control	Realtime audiovisual feedback plus post event debriefing

**SH**	Control	Control

### Rationale for study design

The use of the initial control phase at all sites enables within-site comparison of each intervention with standard care, while the inclusion of Solihull as a control site allows the estimation of the effect of the interventions to be adjusted for changes over time due to changes in other aspects of resuscitation care.

The prospective cohort approach has been chosen because of the risk that the significant learning effect from the feedback amongst the cardiac arrest team could contaminate the results if procedures were individually randomised to the two new interventions or the control. This learning effect would similarly limit the applicability of a cross over trial as a prolonged wash-out period between interventions would be required and even this would not guarantee that any learning from the intervention would not be retained by participants.

### Cardiac arrest team

The cardiac arrest team is activated by contacting a central switchboard (2222). The cardiac arrest team at each site consists of: a Resident Medical Officer (Specialist Trainee year 1-3 or equivalent), critical care doctor, Foundation Year 1 doctor, critical care outreach nurse, senior sister on duty for the hospital and hospital porter. The teams are ALS qualified and work to common practices and procedures. The teams are based at a single site and do not rotate between sites.

### The Q-CPR system

**Figure 1:**
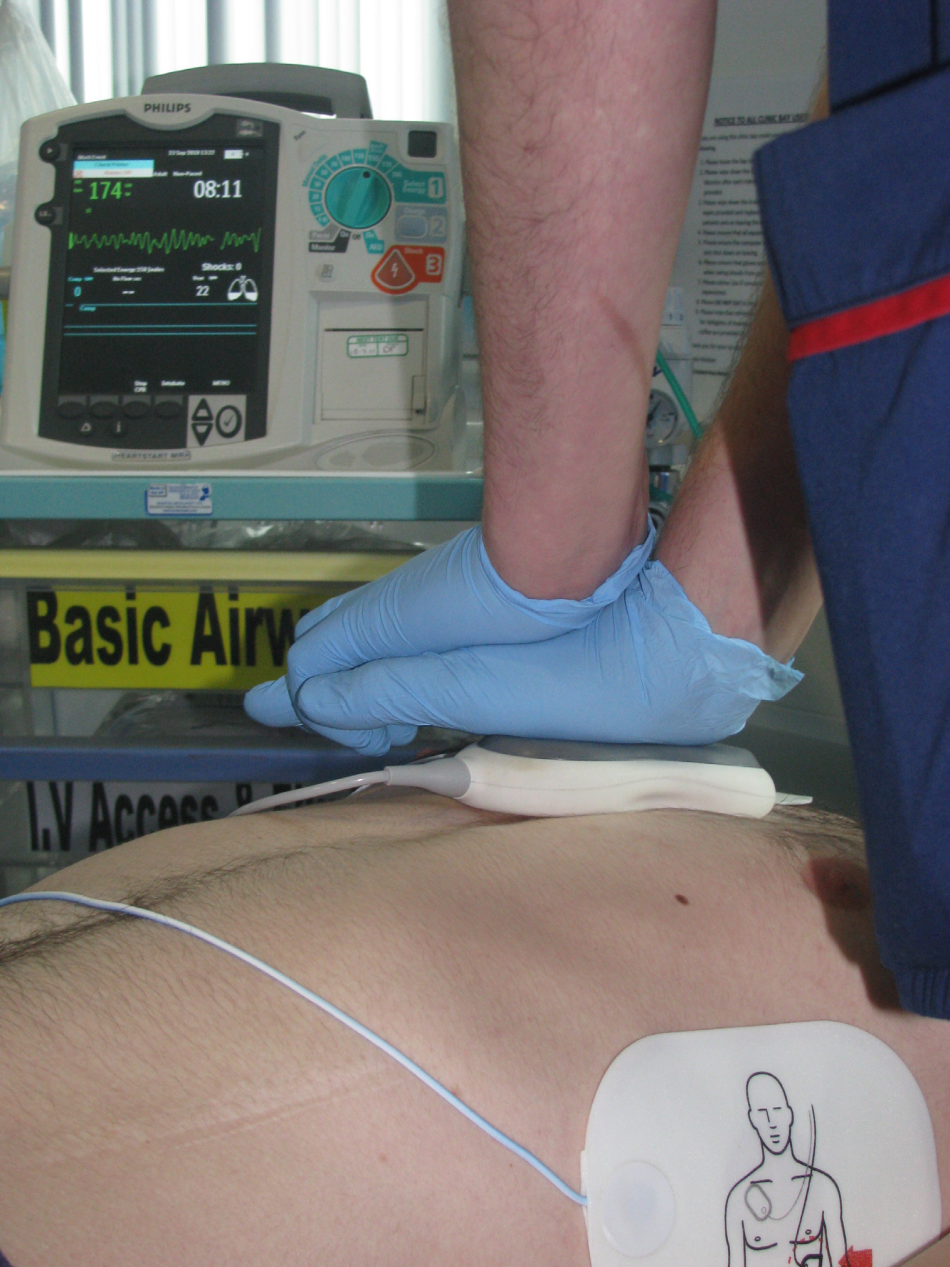
**MRX defibrillator with Q-CPR**.

Existing Trust defibrillators (MRX, Phillips, UK) will be upgraded with Q-CPR technology (Figure 1). Briefly, a 10 × 5 cm device (containing an acceleratometer and force detector) is placed on the patient's chest during resuscitation and measures chest compression parameters. Real-time audio and visual feedback is provided on chest compression rate, depth, incomplete release and duration of interruptions in chest compression. Ventilation rate is calculated from changes in transthoracic impedance measured through the defibrillator pads. The technical details and validation of this device have been previously published [10]. The CPR-review facility captures and exports these data along with continuous ECG, transthoracic impedance and voice recordings of the resuscitation attempt, thus providing audio and detailed visual data about the resuscitation attempt, without the need for potentially cumbersome video recording equipment.

### Real-time feedback

The Q-CPR system will be used to provide real-time audio and visual feedback. Audio feedback comprises spoken messages that are corrective when the performance of CPR deviates from a defined list of parameters. Examples of voice prompts include: 'Blow in more air'; 'Press down deeper'; 'Switch over faster to compressions'. Visual feedback provides information on the adequacy of chest compression depth, compression and ventilation rates and the duration of interruptions in CPR.

### Post-event debriefing

Post-event debriefing will focus on the importance of the quality of CPR and adherence with this during the arrest, the management of the arrest itself (compliance with guidelines), a review of factors leading up to the cardiac arrest and critique of team management. This will be supplemented by a weekly review and debriefing meeting. This will use the approach developed by the Chicago group [17] of reviewing in detail 3-4 cardiac arrests per week - focusing on quality of CPR, treatment decisions and review of the current literature. The results of these meetings will be feedback to the Trust Resuscitation Committee and Trust Safety Committee (Chaired by Dr Woolley PhD, Co-applicant and Executive Director for Governance) to report any health system factors that may require addressing.

### Guidelines change in 2010

The International Liaison Committee for Resuscitation and European Resuscitation Council (UK) published new resuscitation guidelines on 18^th ^October 2010. These were incorporated into clinical practice by the trust in December 2010. The Q-CPR feedback devices will be updated to coincide with the adoption of the new guidelines before the implementation phase begins. The control site (Solihull) will allow for estimation of the interventions to be adjusted for changes due to the new guidelines.

### Data Collection

The following data will be exported from the clinical audit database. No patient identifiable information will be transferred from the clinical database to the research team. Date, time and location of arrest; patient demographics (age, sex); details/quality of emergency 2222 call; suspected cause of cardiac arrest; Q-CPR-download containing information on quality of CPR; resuscitation team composition/qualifications/time since last training; post resuscitation care (duration hospital/ITU length of stay); patient outcomes (survival, cerebral performance category at discharge) and interventions performed (airway, defibrillation, drugs).

### Q-CPR interrogation

Objective CPR performance and ECG data will be downloaded from the study defibrillators and analysed using Q-CPR review software (Phillips, UK). Cardiac rhythm at the start of the resuscitation, as well as before and after defibrillation will be extracted from ECG recordings. The defibrillator will be configured to record CPR quality parameters (compression rate, depth, ventilation rate, no-flow fraction (which represents the fraction of time within a given period that a pulseless patient went without chest compressions) [20]. The duration of pre and post-shock pauses will be calculated manually from the ECG and compression depth waveforms. The time to first shock (for patients initially presenting in a shockable rhythm) will be derived from the time of the cardiac arrest call (recorded at the central switchboard) with the time of shock delivery. The Trust defibrillators are synchronised on a monthly basis with the central switchboard time which achieves a co-efficient of variation in times of < 2%.

### Monitoring of Data Quality

Patient/event data collected by the PDA are checked for fidelity using a detailed periodic re-abstraction process. Initially all data records will be reviewed until an error rate of < 2% is achieved. After this, random sampling of event records will be undertaken by clinical audit staff. This process has been used successfully in multi-centre CPR trials previously [2]. Data requiring manual calculation from the Q-CPR system (rhythm, pre and post shock pauses) will be measured by two researchers and inter-observer agreement calculated and reported. Disagreement will be resolved by re-review and consensus between original 2 researchers.

### Serious Adverse Events (SAEs) and Serious Adverse Device Effects (SADEs)

SAEs and SADEs will be reported to the trial management team if they fulfil the criteria for seriousness, they are potentially related to trial participation, and they are unexpected i.e. the event is not an expected occurrence for patients who have had a cardiac arrest.

### Statistical Analysis

Baseline patient characteristics such as age, sex, aetiology of admission illness, pre-existing illnesses and time of arrest will be summarised for patients at the three hospital sites. Characteristics of patients at the three sites will also be compared. Chi-squared tests will be used for binary variables. Following an assessment of whether they are normally distributed, continuous variable will be compared using linear (ANOVA) models (possibly after transformation) or non-parametric tests.

Binary responses such as the primary outcome of survival for > 20 minutes and the patient-focussed secondary endpoints of return of spontaneous circulation, survival to hospital discharge and cerebral performance at discharge dichotomised to divide patients into two groups will be analysed using a logistic regression approach. The analysis will use data from both phases of the study, with a model that includes a period effect and centre effects. This will allow estimation and testing of hypotheses regarding the effect on the survival rate etc. for the three different intervention arms adjusted for differences between the sites and changes over time. The effect of intervention will also be adjusted for baseline patient characteristics. The effect of intervention will first be fitted as a factor with three levels. If this effect is significant at the p = 0.05 level, further models will be fitted including contrasts to enable pairwise comparisons of the different interventions.

Continuous responses such as the process-focussed secondary outcomes will be analysed using linear regression (ANCOVA) models, possibly after transformation to ensure that the assumption that the residuals from the fitted models are normally distributed is reasonable. As for logistic regression analyses described above, the comparison of the interventions will be adjusted for baseline patient characteristics and differences over time and between the three sites.

## Conclusion

Outcome from cardiac arrest still remains poor. CPR quality is known to significantly influence outcomes from cardiac arrest but despite this, in real life it is often performed sub-optimally. Implementation of CPR quality improvement programme using a real-time CPR feedback device combined with post event debriefing has the potential to improve the quality of CPR compared to current practice. This trial aims to establish whether an improvement in CPR quality will show an improvement in rate of return of spontaneous circulation and other outcome.

## List of abbreviations

CPR: Cardiopulmonary resuscitation; ROSC: Return of spontaneous circulation; TSC: Trial Steering Committee.

## Competing interests

The authors declare that they have no competing interests.

## Authors' contributions

GDP is chief investigator for the trial. The trial protocol was developed by the authors of this paper. GDP prepared the first draft of this summary protocol paper and revised in light of comments from co-investigators. All authors approved the final version of the paper.

## Author information

GDP is a Clinical Professor in Critical Care Medicine. He is Chair of the Resuscitation Committee at Heart of England NHS Trust where he works with Robin Davies (Senior Resuscitation Officer), Sarah Quinton (Lead Nurse Critical Care), Sarah Woolley (Executive Director for Safety and Governance, Fang Gao and Matthew Cooke (Professors). Ben Abella is Clinical Research Director, Center for Resuscitation Science, University of Pennsylvania. Nigel Stallard is Professor of Medical Statistics in the Division of Health Sciences at the University of Warwick

## Supplementary Material

Additional file 1 **Full trial protocol**. Click here for file
