# Supplementary material for: The effect of real-time CPR feedback and post event debriefing on patient and processes focused outcomes: A cohort study: trial protocol
Source: Scand J Trauma Resusc Emerg Med. 2011 Oct 18;19:58. doi: 10.1186/1757-7241-19-58 (PMC3214886; doi:10.1186/1757-7241-19-58)
Supplement: Additional file 1 — Full trial protocol. [file 1757-7241-19-58-S1.PDF]

CPR QUALITY IMPROVEMENT PROGRAMME

PROTOCOL

ISRCTN Number:	TBC
Funding Body:	DH NIHR Research for Patient Benefit
Funders reference:	PB-PG-1207-14246
REC name:	Coventry Research Ethics Committee
REC reference:	09/H1210/65
Ethics Approval Date:	

Version Number	1
Date	23rd April 2009
Stage	Final

Protocol Amendments:

Amendment No.	Date of Amendment	Date of Approval

HEART of ENGLAND NHS Foundation Trust
NHS National Institute for Health Research
Warwick Medical School CLINICAL TRIALS UNIT

CONTACT NAMES AND NUMBERS

Role	Name, address, telephone
**Sponsor:**	Heart of England NHS Foundation Trust<br>Elizabeth Adey<br>Bordesley Green East<br>Birmingham<br>B9 5SS<br>Tel: 0121 424 1633<br>Email: Elizabeth.Adey@heartofengland.nhs.uk
**Chief Investigator:**	Dr Gavin Perkins<br>Warwick Medical School Clinical Trials Unit<br>University of Warwick<br>Coventry<br>CV4 7AL<br>Tel: 0121 424 2966 (Dawn Hill- Heartlands)<br>Tel: 024 761 50925 (Nicola Morrow - Warwick)<br>Fax: +44 (0) 871 2530597<br>Email: g.d.perkins@warwick.ac.uk
**Trial Co-ordinator:**	Teresa Melody<br>Academic Department of Critical Care, Anaesthesia and Pain<br>Birmingham Heartlands Hospital<br>Birmingham<br>B9 5SS<br>Tel: 07966 286453<br>Fax: 0121 424 2971<br>Email: Teresa.Melody@heartofengland.nhs.uk

TRIAL STEERING COMMITTEE

Professor Matthew Cooke (Chair)
Professor of Emergency Medicine and Clinical Systems improvement
Warwick Medical School and Heart of England NHS Foundation Trust

Dr Gavin Perkins
Associate Clinical Professor Critical Care and Resuscitation
Warwick Medical School and Heart of England NHS Foundation Trust

Professor Fang Gao
Warwick Medical School and Heart of England NHS Foundation Trust

Mr Robin Davies
Lead Resuscitation Office, Heart of England NHS Foundation Trust

Dr Sarah Woolley
Executive Director for Governance
Heart of England NHS Trust

Professor Nigel Stallard
Professor of Medical Statistics
University of Warwick Medical School

Dr Ben Abella
Associate Professor and Director of Clinical Research
University of Pennsylvania, Philadelphia, US

Ms Sarah Quinton
Lead Critical Care Outreach Nurse
Heart of England NHS Foundation Trust

Mr John Long
Royal Life Saving Society

TABLE OF CONTENTS **PAGE**

	TABLE OF CONTENTS	4
1.	BACKGROUND	6
2.	STUDY DESIGN	7
2.1	Research Question	7
2.2	Hypothesis	7
2.3	Study design	7
2.4	Rationale for study design	8
2.5	Cardiac arrest team	8
2.6	Equipment	9
2.7	Intervention	9
2.8	Eligibility	9
2.9	Inclusion criteria	10
2.10	Exclusion criteria	10
2.11	Outcomes	10
2.12	Outcome definitions / justification	11
2.13	Sample size estimation	11
2.14	Feasibility	12
3.	ANALYSIS PLAN	12
4.	STUDY CONDUCT	13
4.1	Ethical considerations	13
4.2	Good Clinical Practice	13
5.	DATA MANAGEMENT	13
5.1	Data protection act	13
5.2	Clinical database	14
5.3	Schedule of data collection	14
5.4	Q-CPR interrogation	14
5.5	Monitoring of data quality	15
5.6	Protection against bias	15
5.7	Database	15
5.8	Data Storage	15
5.9	Archiving	15

6. START AND END OF STUDY 16
7. TRIAL ORGANISATION AND OVERSIGHT 18
7.1 Ethical conduct of the trial 18
7.2 Sponsor 18
7.3 Indemnity 18
7.4 Administration 18
7.5 Trial Management Group (TMG) 19
7.6 Trial Steering Committee (TSC) 19
7.7 Essential Documentation 19
8. DISSEMINATION AND PUBLICATION 19
9. FINANCIAL SUPPORT 20
10. REFERENCES 20
11. APPENDICES 22

1. BACKGROUND

Each year approximately 30-35,000 people sustain a cardiac arrest in hospitals in the UK. National audits in the UK and US report an initial survival rate of 50-60%[1, 2]. Morbidity and risk of death are high in the first few days after cardiac arrest, however, after this high risk period has passed, the majority (80%) of people that survive cardiac arrest and are alive at hospital discharge relatively free from on-going morbidity and alive at one year.

The International Liaison Committee for Resuscitation (ILCOR) have developed evidence based guidelines for resuscitation which are used across NHS trusts[3]. However these can only improve outcomes if they are successfully implemented into clinical practice[4].

The importance of the quality of CPR has been reinforced in a series of observational studies in humans. Chest compression depth[5, 6]; rate[7]; ventilation rate[8] and duration of pre-shock pauses[6, 9] have all been shown to influence the likelihood of a successful resuscitation attempt. Despite these compelling data, observational studies during real life resuscitation attempts consistently demonstrate sub-optimal implementation of resuscitation guidelines in practice[10,11].

The integration of real-time audio/visual feedback during actual CPR improves the quality of CPR in pre-hospital resuscitation attempts[5], but it's effect in hospital has been less clearly demonstrated[12]. The use of post event debriefing in simulator based CPR training significantly improves team performance and patient outcomes[13,14] and is widely used in military and aviation practices for improving future performance. Junior doctors have also reported feeling un-prepared and concerned about managing cardiac arrest and have called for more feedback on their performance [15]. Dr Ben Abella (University of Pennsylvania School of Medicine) has developed a system of post-event debriefing of the cardiac arrest team. They went on to show that post-event feedback was associated with improved: knowledge; adherence to guidelines; quality of CPR and a significantly increase in survival (44% to 60%, P=0.03)[16].

This promising study has some limitations and it is unclear if these findings could be directly extrapolated to UK practice. Firstly, the historical control group preceded a major change in resuscitation guidelines (Guidelines 2005), so it is unclear if the improvement in survival was contaminated by the change in practice. Secondly, the casemix in this study are significantly different from UK practice (63% of arrests occurred in a critical care area as opposed to 11% in our own Trust and 15% nationally). Thirdly, CPR feedback technology was already in widespread use with the control cohort, which is rare in the UK at present.

In the recent ILCOR knowledge gaps and research prioritisation exercise, the paucity of evidence in this area was identified and the need for further research on feedback during and after CPR was prioritised[17].

The aim of this project is to test whether implementation of a CPR quality initiative (comprising of real-time CPR feedback technology supplemented with post-event debriefing) affect patient and process focused outcomes.

2. STUDY DESIGN

This work bridges the domains of service improvement and research as defined in the NRES Guidance 2007 on differentiating audit, research and service evaluation. It is being submitted for ethical review in light of the overlap with research and development activity.

The Heart of England NHS Trust are committed to monitoring process and outcomes from cardiac arrest. The Trust commitment to this is defined in the Resuscitation Policy. The policy addresses the Trust's statutory requirements to monitor cardiac arrest rates and outcomes (Health Service Circular HSC2000/028) and other guidance documents (Standards for Clinical Practice and Training - Royal College of Physicians).

The Trust wish to introduce audio/visual prompts and post event debriefing on the quality of CPR and patient outcomes. Our desire to undertake this evaluation in a systematic manner and share findings with the wider healthcare community overlaps service development with research activity.

2.1 Research Question

In hospitalised adult patients in whom resuscitation from cardiac arrest is attempted, will a CPR quality improvement programme compared to current practice, change patient and process focused outcomes?

2.2 Hypothesis

Implementing a CPR quality improvement initiative will improve the rate of return of spontaneous circulation and other outcomes in patients sustaining cardiac arrest.

2.3 Study design

This study will be a prospective, cohort study. The study will evaluate two interventions: real-time audio-visual feedback during CPR and real-time audio-visual feedback during CPR plus post event feedback using the Q-CPR system (described below). We have not included a 3rd arm (post event feedback alone) as this would be unlikely to be used in clinical practice as to be able to provide this model of post event feedback requires the use of the Q-CPR equipment, which incorporates the real-time audiovisual feedback facility.

The study will be divided into 2 phases. During the first phase, baseline data will be collected at each site. During the 2nd phase, real-time feedback phase will be implemented at one site (Good Hope-GH) and real-time audiovisual feedback plus post event debriefing at one site (Heartlands-BHH). The remaining site (Solihull-SH) will act as the control site during both interventions. The purpose of the control site is to exclude any Trust-wide changes in care as potential explanations for any changes in outcomes at Heartlands/Good Hope.

Site	Phase 1	Phase 2
GHH	Control	Realtime audiovisual feedback
BHH	Control	Realtime audiovisual feedback plus post event debriefing
SH	Control	Control

2.4 Rationale for study design

The use of the initial control phase at all sites enables within-site comparison of each intervention with standard care, while the inclusion of Solihull as a control site allows the estimation of the effect of the interventions to be adjusted for changes over time due to changes in other aspects of resuscitation care.

The prospective cohort approach has been chosen because of the risk that the significant learning effect from the feedback amongst the cardiac arrest team could contaminate the results if procedures were individually randomised to the two new interventions or the control. This learning effect would similarly limit the applicability of a cross over trial as a prolonged wash-out period between interventions would be required and even this would not guarantee that any learning from the intervention would not be retained by participants.

2.5 Cardiac arrest team

The cardiac arrest team is activated by contacting a central switchboard (2222). The cardiac arrest team at each site consists of: a Resident Medical Officer (ST1-3 or equivalent who acts as team leader), critical care doctor, Foundation Year 1 doctor, critical care outreach nurse, senior sister on duty for the hospital and hospital porter. The teams are ALS qualified and work to common practices and procedures. The teams are based at a single site and do not rotate between sites.

2.6 Equipment

Existing Trust defibrillators (MRX, Phillips, UK) will be upgraded with Q-CPR technology. Briefly, a 10 x 5 cm device (containing an accelerometer and force detector) is placed on the patient's chest during resuscitation and measures chest compression parameters. Real-time audio and visual feedback is provided on chest compression rate, depth, incomplete release and duration of interruptions in chest compression. Ventilation rate is calculated from changes in transthoracic impedance measured through the defibrillator pads. The technical details and validation of this device have been previously published[10]. The CPR-review facility captures and exports these data along with continuous ECG, transthoracic impedance and voice recordings of the resuscitation attempt, thus providing audio and detailed visual data about the resuscitation attempt, without the need for potentially cumbersome video recording equipment.

2.7 Intervention

Realtime feedback: The Q-CPR system will be used to provide real-time audio and visual feedback. Audio feedback comprises spoken messages that are corrective when the performance of CPR deviates from a defined list of parameters. Examples of voice prompts include: 'Blow in more air'; 'Press down deeper'; 'Switch over faster to compressions'. Visual feedback provides information on the adequacy of chest compression depth, compression and ventilation rates and the duration of interruptions in CPR.

Post-event debriefing: An immediate post-event debriefing will be led by the critical care outreach team. This will focus on the importance of the quality of CPR and adherence with this during the arrest, the management of the arrest itself (compliance with guidelines), a review of factors leading up to the cardiac arrest and critique of team management. This will be supplemented by a weekly review and debriefing meeting. This will use the approach developed by the Chicago group[16] of reviewing in detail 3-4 cardiac arrests per week – focusing on quality of CPR, treatment decisions and review of the current literature. The results of these meetings will be feedback to the Trust Resuscitation Committee and Trust Safety Committee (Chaired by Dr Woolley PhD, Co-applicant and Executive Director for Governance) to report any health system factors that may require addressing.

2.8 Eligibility

Consecutive hospitalized adult patients who sustain a cardiac arrest during their hospital stay will be eligible for inclusion in the study according to the following criteria.

2.9 Inclusion criteria

Age $\geq$ 18

Cardiac arrest where resuscitation is attempted (defined as loss of a pulse requiring the delivery of chest compressions).

2.10 Exclusion criteria

Patient has a Do-not-attempt resuscitation order written and docummented in their medical records

Cardiac Arrest not attended by a resuscitation team

Out –of-hospital cardiac arrest

Previous participation in this study

2.11 Outcomes

Primary outcome

i. Survived event (defined as sustained (> 20 minutes) return of spontaneous circulation)

Secondary outcomes – patient focused

i. Any return of spontaneous circulation

ii. Survival to hospital discharge

iii. Cerebral performance category of patients at discharge

Secondary outcomes – process focused

i. Quality of CPR

a. quality of 2222 call

b. chest compression depth

c. chest compression rate

d. no-flow time, no-flow time adjusted

e. duration of pre-shock and post shock pause

f. ventilation rate

g. time to first shock (if initial rhythm VF/VT).

h. appropriate decision to shock

ii. Team factors

a. CPR knowledge amongst the cardiac arrest team

b. Confidence / Preparedness

2.12 Outcome definitions / justification

The main study outcomes will be reported in accordance with the Utstein template for in-hospital resuscitation[18] and guidelines for reporting studies on quality of CPR[19]. The following standardised definitions will be used: Survived event - defined as sustained (> 20 minutes) return of spontaneous circulation. Any return of spontaneous circulation (return of a palpable pulse at any point following commencement of CPR); survival to hospital discharge (alive and discharged from the admitting hospital). Neurological outcome will be assessed by Cerebral performance category (CPC 1 is good cerebral performance; CPC 2 moderate cerebral disability; CPC 3 severe cerebral disability; CPC 4 coma/vegetative state; and CPC 5 brain death)[18]. CPR quality variables will be defined according to standard definitions [19]. CPR knowledge amongst the cardiac arrest team will be measured by multiple choice questions, taken from the Resuscitation Council (UK) database of validated questions at the end of each control and intervention period. Confidence / preparedness amongst cardiac arrest team members will be measured using the questionnaire developed by Hayes[15].

2.13 Sample size estimation

The sample size estimation is based on a baseline return of spontaneous circulation rate of 44% (from the last 12 month audit at the Trust). To predict a 16% absolute improvement in ROSC rate (as seen in the Edelson et al[16] study which had a similar baseline rate as our Trust) 152 patients will be required in each arm to achieve 80% power at a significance level of 0.05.

Secondary outcomes: Based on the data from the Edelson study and our own preliminary studies (n=6); the study will have sufficient power (80%) at a significance level of 0.01 to detect the following improvements in CPR quality performance data (all figures are relative changes from baseline): chest compression depth (8%); ventilation rate (12%); no flow fraction (18%); appropriate decision to shock (15%).

2.14 Feasibility

The number of documented cardiac arrests across the 3 in-patient hospital sites was recorded as 620 in 2007 (Heartlands 297; Solihull 168; Good hope 186). We propose to run the control phase and intervention phase at Heartlands and Good Hope for 13 months each. Patients arresting on more than one occasion were not included in this audit. Cardiac arrest event data are used as a quality indicator by the Trust, so based on our current experience we anticipate that the data informing the primary outcome of the study (survived event) will be complete. Although we anticipate capturing all eligible cardiac arrests, we have based our recruitment windows assuming an 80% enrollment rate at the smallest centre (Solihull).

3. ANALYSIS PLAN

Baseline patient characteristics such as age, sex, aetiology of admission illness, pre-existing illnesses and time of arrest will be summarised for patients at the three hospital sites. Characteristics of patients at the three sites will also be compared. Chi-squared tests will be used for binary variables. Following an assessment of whether they are normally distributed, continuous variable will be compared using linear (ANOVA) models (possibly after transformation) or non-parametric tests.

Binary responses such as the primary outcome of survival for > 20 minutes and the patient-focussed secondary endpoints of return of spontaneous circulation, survival to hospital discharge and cerebral performance at discharge dichotomised to divide patients into two groups will be analysed using a logistic regression approach. The analysis will use data from both phases of the study, with a model that includes a period effect and centre effects. This will allow estimation and testing of hypotheses regarding the effect on the survival rate etc. for the three different intervention arms adjusted for differences between the sites and changes over time. The effect of intervention will also be adjusted for baseline patient characteristics. The effect of intervention will first be fitted as a factor with three levels. If this effect is significant at the p=0.05 level, further models will be fitted including contrasts to enable pairwise comparisons of the different interventions.

Continuous responses such as the process-focussed secondary outcomes will be analysed using linear regression (ANCOVA) models, possibly after transformation to ensure that the assumption that the residuals from the fitted models are normally distributed is reasonable. As for logistic regression analyses described above, the comparison of the interventions will be adjusted for baseline patient characteristics and differences over time and between the three sites.

4. STUDY CONDUCT

4.1 Ethical considerations

Participation in the trial will commence following the onset of cardiac arrest and finish following return of spontaneous circulation or discontinuation of resuscitation efforts. Patients will be resuscitated according to current Resuscitation Council (UK) guidelines. The “intervention” in the trial (the use of audio/visual prompts and post event debriefing) serve as a mechanism to improve adherance with recommended best practice. The device (Q-CPR) which will record quality of CPR parameters has CE mark (CE 0123) and will be being used within its current licence and therefore does not require separate registration of the trial with the MHRA (Devices).

The unpredictable and immediately incapacitating nature of cardiac arrest (sudden loss of consciousness) means that it will not be possible to obtain prospective informed consent from participants. Because the trial recruits in an emergency situation, it will also not be possible to obtain assent from their relatives before enrolment. The trial will be subject to the requirements of the Mental Capacity Act 2005 and will require approval from an appropriate Research Ethics Committee (REC) in England.

Process and outcome data are routinely recorded on the Trust clinical CPR audit database. Data for analysis for research purposes will be extracted and anonomised from this database.

Regretably the nature of the emergency in question mean that the majority (>80%) of resuscitation attempts will be unsuccessful. Our experience of approaching relatives in the hours and days after a cardiac arrest has been that this causes distress and confusion at a time when they are already burdended by either the loss of a loved one or shock of a sudden deterioation in health status. Our assessment of the burdens of approaching relatives or patients for consent to use anonymised data which are already routinely collected outweigh the benefits.

4.2 Good Clinical Practice

The study will be carried out in accordance with the Medical Research Council (MRC) Good Clinical Practice Guidelines and applicable UK legislation.

5. DATA MANAGEMENT

5.1 Data protection act

The management of data collected in this study will comply with the Data Protection Act (1998) and Trust policies for the management of data.

5.2 Clinical database

The Trust is required to monitor process and outcomes from cardiac arrest (Health Service Circular HSC2000/028 and Standards for Clinical Practice and Training - Royal College of Physicians).

These data are recorded on the Trust resuscitation database which is managed by the Resuscition Service. Data stored on the Trust database are managed in accordance with Trust policy.

Data for use for research purposes will be exported in anonimised form with no patient identifiable information.

5.3 Schedule of data collection

The following data will be exported from the clinical audit database. No patient identifiable information will be transferred from the clinical database to the research team.

- Date, time and location of arrest
- Patient demographics (age, sex)
- Details / quality of emergency 2222 call
- Suspected cause of cardiac arrest
- QCPR-download containing information on quality of CPR
- Resuscitation team composition / qualifications / time since last training
- Post resuscitation care – duration hospital / ITU lenght of stay.
- Patient outcomes – survival, cerebral performance category at discharge
- Interventions performed (airway, defibrillation, drugs)

5.4 Q-CPR interrogation

Objective CPR performance and ECG data will be downloaded from the study defibrillators and analysed using Q-CPR review software (Phillips, UK). Cardiac rhythm at the start of the resuscitation, as well as before and after defibrillation will be extracted from ECG recordings. The defibrillator will be configured to record CPR quality parameters (compression rate, depth, ventilation rate, no-flow fraction (which represents the fraction of time within a given period that a pulseless patient went without chest compressions) in 30 second segments and as aggregate mean and standard deviation values over the duration of the cardiac arrest (from initiation of CPR until ROSC) [19]. The duration of pre and post-shock pauses will be calculated manually from the ECG and compression depth waveforms. CPR quality will be deemed within the target range for each 30 second segment according to the consensus conference guidelines if the compression rate is between 90-120 min, depth > 38mm; ventilation rate < 15 minute[19]. The time to first shock (for patients initially presenting in a shockable rhythm) will be derived from the time of the cardiac arrest call (recorded at the central switchboard) with the time of shock delivery. The Trust defibrillators are synchronised

on a monthly basis with the central switchboard time which achieves a co-efficient of variation in times of <2%.

5.5 Monitoring of data quality

Patient / event data collected by the PDA are checked for fidelity using a detailed periodic re-abstraction process. Initially all data records will be reviewed until an error rate of < 2% is achieved. After this, random sampling of event records will be undertaken by clinical audit staff. This process has been used successfully in multi-centre CPR trials previously[2]. Data requiring manual calculation from the Q-CPR system (rhythm, pre and post shock pauses) will be measured by two researchers and inter-observer agreement calculated and reported. Disagreement will be resolved by re-review and consensus between original 2 researchers.

5.6 Protection against bias

It is not possible to blind either the cardiac arrest team or investigator team from the study intervention. The following steps will be taken to protect against bias. The primary and secondary patient focused outcomes are objective outcomes that cannot be influenced by knowledge of the treatment allocation. The CPR performance and electrocardiographic data are collected electronically directly from the study defibrillators after resuscitation attempts and are therefore not subject to bias. Researchers measuring parameters requiring interpretation from Q-CPR (% correct shock decisions, pre and post-shock pauses) will be blinded from knowing whether the data are from the control or intervention site.

5.7 Database

A database will be set up by the research team and all specifications (ie database variables, validation checks, screens) will be agreed between the programmer, statistician and trial co-ordinator.

5.8 Data Storage

All essential documentation and trial records will be stored by Heart of England NHS Trust in conformance with the applicable regulatory requirements and access to stored information will be restricted to authorised personnel.

5.9 Archiving

Trial documentation and data will be archived for at least five years after completion of the trial. ahering to the trust policy and procedure documentation for archiving which is implemented by the Trusts Research and Development Governance Manager

6. START AND END OF STUDY

It is anticipated that preparatory work for the trial will commence in June 2009. Data collection for the control period will start three months later. Data collection will continue for 26 months. The trial timetable is summarised in the diagram below.

The trial will be stopped prematurely if:

- Mandated by the Ethics Committee
- Funding for the trial ceases

The Main Research Ethics Committee (MREC) will be notified in writing if the trial has been concluded or terminated early.

Project plan / timelines

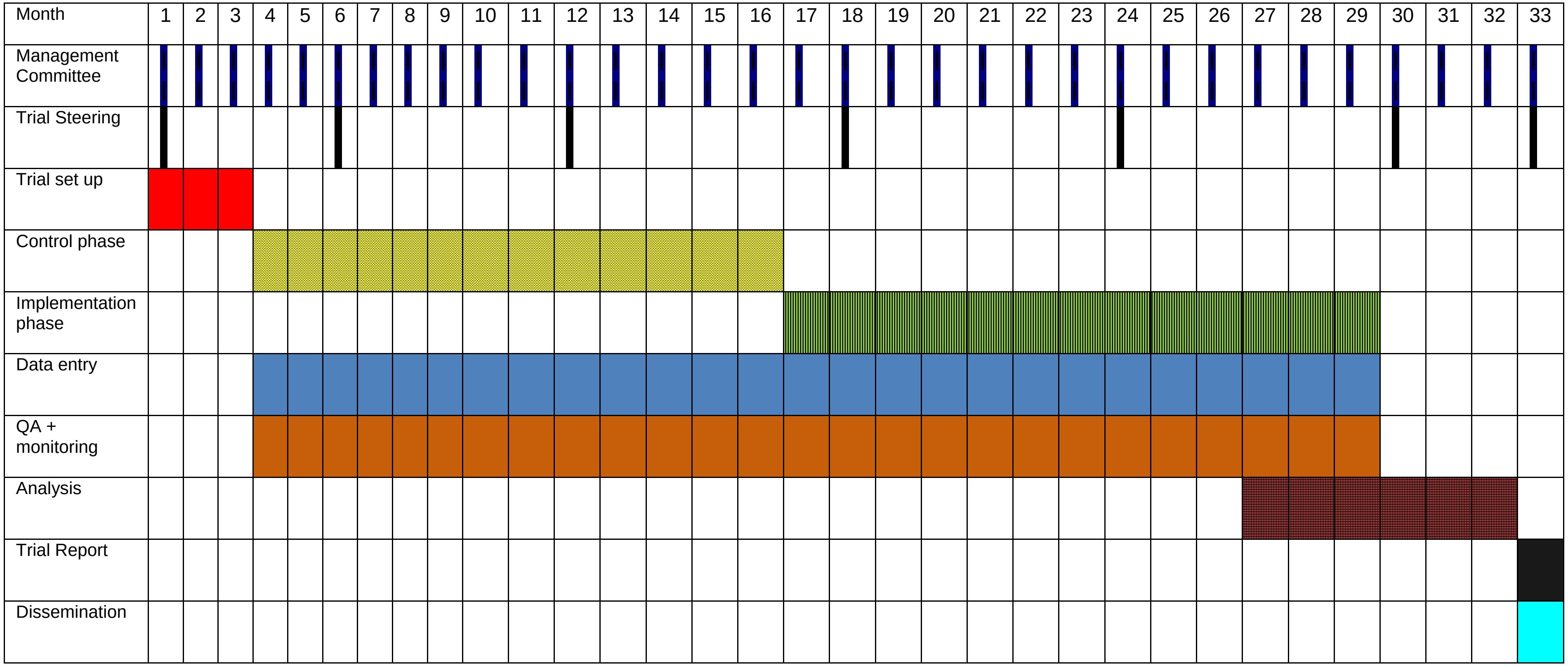

7. TRIAL ORGANISATION AND OVERSIGHT

7.1 Ethical conduct of the trial

The study will be performed in accordance with ethical principles that have their origin in the Declaration of Helsinki and are consistent with MRC Good Clinical Practice and applicable regulatory requirements.

7.2 Sponsor

Heart of England NHS Foundation Trust will act as sponsors for the trial

7.3 Indemnity

NHS indemnity covers NHS staff. NHS bodies carry this risk themselves or spread it through the Clinical Negligence Scheme for Trusts, which provides unlimited cover for this risk.

The University of Warwick will indemnify their staff via the University Insurance Policy.

7.4 Administration

The project will be managed and over seen by the Chief Investigator.

Teresa Melody (Research Manager) will be responsible for the day to day management of trial staff and for overview of the project.

A Trial manager / Research assistant will be appointed and will act as the central contact for the study and will be responsible for day to day trial management, training and liaison with staff. They will be employed for the full duration of the project.

Robin Davies (Senior Resuscitation Officer) will act as the link between the Trust Resuscitation Service and research team. He and his team are responsible for the ownership, maintenance and security of the Trust clinical outcome database. His team will support the delivery of post event debriefing during the second phase of the trial.

Sarah Quinton (Lead Nurse Critical Care Outreach) will provide liaison between the project team and the resuscitation teams and will contribute to the weekly debriefing meetings and will work on the project 1 hour per week from the start of the project until the end of patient recruitment.

Professor Stallard will provide statistical oversight for the duration of the project. During the final stages of the project an additional statistican (0.66 FTE) will be required to undertake the data analysis and modelling.

The Heart of England NHS Foundation Trust Research and Developement department, represented by Liz Adey (manager) will oversee the financial management of the trial and as Sponsors will ensure that statutory requirements are fulfilled.

7.5 Trial Management Group (TMG)

The trial management group will comprise the CI, Research Manager, Research Assistant, Senior Resuscitation Officer and Critical Care Outreach team. Regular (initially weekly) meetings will be held to discuss problems and monitor progress.

7.6 Trial Steering Committee (TSC)

The trial will be guided by a group of respected and experienced clinicians as well as a ‘lay’ representative. The TSC will chaired by Professor Matthew Cooke. Face to face meetings will be held at regular intervals determined by need but not less than once a year. Routine business is conducted by email, post or teleconferencing.

The Steering Committee, in the development of this protocol and throughout the trial will take responsibility for:

- Major decisions such as a need to change the protocol for any reason
- Monitoring and supervising the progress of the trial
- Reviewing relevant information from other sources
- Considering recommendations from the DMEC
- Informing and advising on all aspects of the trial

7.7 Essential Documentation

A Trial Master file will be set up and held securely at the co-ordinating centre.

8. DISSEMINATION AND PUBLICATION

The findings from this research will be shared in several ways. The results will be initially presented to a meeting of investigators, collaborators and patients. Opportunities to present the findings at regional and national medical, emergency medicine, anaesthetic and critical care meetings will be sought and a summary of the research findings will be placed on the National Electronic Library for Health.

It is also our intention to present the data at international meetings of researchers with open access abstracts on-line e.g. Resuscitation Science Symposium. We will submit the main and supplementary papers generated from this research to Medline indexed journals thereby creating a stable archive of peer-reviewed research publications ensuring the permanent preservation of the research findings. A condition of the grant supporting this programme is that papers must be made freely available by publishers by six months after first publication. This will both secure a searchable compendium of these publications and make the results of our research readily accessible to the public, health care professionals and scientists alike.

A report of the study findings will be sent to INVOLVE registery. In addition, the most significant results will be communicated to the public through press releases. Where appropriate, research details will also be posted on Institutional websites available to the general public. Clinically relevant information will be publicised through the Research & Development Office of the Heart of England NHS trust who have an excellent record of fostering communication between clinicians, the media and general public.

9. FINANCIAL SUPPORT

The study is supported by the Department of Health National Institute for Health Research, Research for Patient Benefit Programme PB-PG-1207-14246

10. REFERENCES

1.Gwinnutt CL, Columb M, Harris R. Outcome after cardiac arrest in adults in UK hospitals: effect of the 1997 guidelines. Resuscitation. 2000 2000 Oct;47(2):125-35.

2.Nadkarni VM, Larkin GL, Peberdy MA, et al. First documented rhythm and clinical outcome from in-hospital cardiac arrest among children and adults. JAMA. 2006 Jan 4;295(1):50-7.

3.Deakin CD, Nolan JP, Perkins GD, et al. Advanced Life Support Guidelines for the UK. 2005. p. 15-30.

4.Chamberlain DA, Hazinski MF. Education in resuscitation: an ILCOR symposium: Utstein Abbey: Stavanger, Norway: June 22-24, 2001. Circulation. 2003 Nov 18;108(20):2575-94.

5.Kramer-Johansen J, Myklebust H, Wik L, et al. Quality of out-of-hospital cardiopulmonary resuscitation with real time automated feedback: a prospective interventional study. Resuscitation. 2006;71(3):283-92.

6.Edelson DP, Abella BS, Kramer-Johansen J, et al. Effects of compression depth and pre-shock pauses predict defibrillation failure during cardiac arrest. Resuscitation. 2006 Nov;71(2):137-45.

7.Abella BS, Sandbo N, Vassilatos P, et al. Chest compression rates during cardiopulmonary resuscitation are suboptimal: a prospective study during in-hospital cardiac arrest. Circulation. 2005 Feb 1;111(4):428-34.

8.Aufderheide TP, Lurie KG. Death by hyperventilation: a common and life-threatening problem during cardiopulmonary resuscitation. Crit Care Med. 2004 Sep;32(9 Suppl):S345-S51.

9.Eftestol T, Sunde K, Steen PA. Effects of interrupting precordial compressions on the calculated probability of defibrillation success during out-of-hospital cardiac arrest. Circulation. 2002 May 14;105(19):2270-3.

10.Abella BS, Alvarado JP, Myklebust H, et al. Quality of cardiopulmonary resuscitation duing in-hospital cardiac arrest. JAMA. 2005 Jan 19;293(3):305-10.

11.Wik L, Kramer-Johansen J, Myklebust H, et al. Quality of cardiopulmonary resuscitation during out-of-hospital cardiac arrest. JAMA. 2005 Jan 19;293(3):299-304.

12.Abella BS, Edelson DP, Kim S, et al. CPR quality improvement during in-hospital cardiac arrest using a real-time audiovisual feedback system. Resuscitation. 2007;73(1):54-61.

13.Scherer LA, Chang MC, Meredith JW, et al. Videotape review leads to rapid and sustained learning. Am J Surg. 2003 Jun;185(6):516-20.

14.Townsend RN, Clark R, Ramenofsky ML, et al. ATLS-based videotape trauma resuscitation review: education and outcome. J Trauma. 1993 Jan;34(1):133-8.

15.Hayes CW, Rhee A, Detsky ME, et al. Residents feel unprepared and unsupervised as leaders of cardiac arrest teams in teaching hospitals: a survey of internal medicine residents. Crit Care Med. 2007 Jul;35(7):1668-72.

16.Edelson DP, Litzinger B, Arora V, et al. Resuscitation with actual performance integrated debriefing (RAPID) improves CPR quality and initial patient survival. Resuscitation Science Symposium. 2008;A61:29.

17.Gazmuri RJ, Nolan JP, Nadkarni VM, et al. Scientific knowledge gaps and clinical research priorities for cardiopulmonary resuscitation and emergency cardiovascular care identified during the 2005 International Consensus Conference on ECC and CPR Science with Treatment Recommendations A Consensus Statement from the International Liaison Committee on Resuscitation; the American Heart Association Emergency Cardiovascular Care Committee; the Stroke Council; and the Cardiovascular Nursing Council. Resuscitation. 2007;75(3):400-11.

18.Jacobs I, Nadkarni V, Bahr J, et al. Cardiac arrest and cardiopulmonary resuscitation outcome reports: update and simplification of the Utstein templates for resuscitation registries. A statement for healthcare professionals from a task force of the international liaison committee on resuscitation (American Heart Association, European Resuscitation Council, Australian Resuscitation Council, New Zealand Resuscitation Council, Heart and Stroke Foundation of Canada, InterAmerican Heart Foundation, Resuscitation Council of Southern Africa). Resuscitation. 2004;63(3):233-49.

19.Kramer-Johansen J, Edelson DP, Losert H, et al. Uniform reporting of measured quality of cardiopulmonary resuscitation (CPR). Resuscitation. 2007 Sept;74(3):406-17.

20.Gage H, Kenward G, Hodgetts TJ, et al. Health system costs of in-hospital cardiac arrest. Resuscitation. 2002 Aug;54(2):139-46.

11. APPENDICES

<<Appendices may include: sample questionnaires e.g. QOL, toxicity grading system, details information on treatment regimens, drug supply details, summary of product characteristics>>
